# Spontaneous Subdiaphragmatic Hemorrhage From an Aneurysm of Inferior Phrenic Artery

**DOI:** 10.14309/crj.0000000000001395

**Published:** 2024-06-21

**Authors:** Venkata Siva Krishna Kumar Pulivarthi, Yamini Katamreddy, Sai Swarupa Vulasala, Jayabharath Onteddu, Saikiran Mandyam, Nirmal Onteddu

**Affiliations:** 1 Department of Internal Medicine, Creighton University School of Medicine, Omaha, NE; 2 Department of Internal Medicine, West Anaheim Medical Center, Anaheim, CA; 3 Department of Internal Medicine/Radiology, East Carolina Health Medical Center, Greenville, NC; 4 Department of Internal Medicine, Viswabharathi Medical College, Andhra Pradesh, India; 5 Department of Internal Medicine, Southeast Health, Dothan, AL; 6 Department of Internal Medicine, Flowers Hospital, Dothan, AL

**Keywords:** Inferior phrenic artery, visceral aneurysms, pseudoaneurysms, segmental arterial mediolysis

## Abstract

Inferior phrenic artery (IPA) aneurysms are the rarest type of visceral aneurysms. It usually occurs secondary to trauma, surgery, or as a complication of pancreatitis. In addition, it can be a manifestation of underlying systemic pathology such as vasculitis, collagen vascular disorders, sepsis, or segmental arterial mediolysis. It can be associated with hypertension in 43% of cases. The presentation of IPA aneurysm is nonspecific with abdominal pain, melena, hematochezia, and anemia. The ruptured and actively bleeding aneurysm can lead to hemorrhagic shock, and immediate management is required with angiography and endovascular embolization with coil or gel foam or stent etc. Inaccessible locations are reached with surgical intervention, but it is associated with high morbidity and mortality. We here report a rare case of spontaneously ruptured IPA pseudoaneurysm extending from the posterior mediastinum to the subdiaphragmatic area and managed with coil and gel foam embolization.

## INTRODUCTION

The posterior mediastinum is bounded anteriorly by the pericardium and great vessels, posteriorly by the prevertebral fascia, and laterally by the pleura.^1^ It constitutes the esophagus, aorta, thoracic duct, distal azygous-hemiazygous venous system, paravertebral soft tissues, and paraspinal nervous system. Any case presenting with posterior mediastinal mass shall have a differential diagnosis of neurogenic tumors, foregut cysts, sarcoma, aneurysms, esophageal neoplasms, intrathoracic goiter, mediastinal pseudocysts, and lymphoma.^1,2^ Around 10% of masses are of vascular origin,^3^ of which inferior phrenic artery (IPA) pseudoaneurysm is a rare one that comes into the category of visceral aneurysms.^4^ Pseudoaneurysms are outpouching from the arterial blood vessels and occur due to a breach in the vessel. They are considered false aneurysms as they are contained by the tunica adventitia or by the wall formed by fibrin and platelet aggregates.^5^ Whereas true arterial aneurysms have all the layers of the wall as the running blood vessel. They are seen in any part of the aorta, femoral artery, carotid artery, visceral arteries, etc. Visceral artery pseudoaneurysm can be present along the celiac artery and its branches (39%), hepatic (39%), splenic (18%), and superior mesenteric artery (4%).^6^ IPA pseudoaneurysm can occur after any catheter-based interventions, as a complication of pancreatitis,^4^ trauma, and surgeries like coartectomy,^7,8^ and gastrectomy.^4,9^ The other possible causes include sepsis, vasculitis (Polyarteritis nodosa, Takayasu arteritis, Behcet syndrome, and Henoch-Schnolein purpura), collagen vascular diseases, and segmental arterial mediolysis (SAM).^10^

We report a very rare case of spontaneous subdiaphragmatic hemorrhage from a pseudoaneurysm of the IPA, later managed with gel foam and coil embolization.

## CASE REPORT

**Figure 1.**
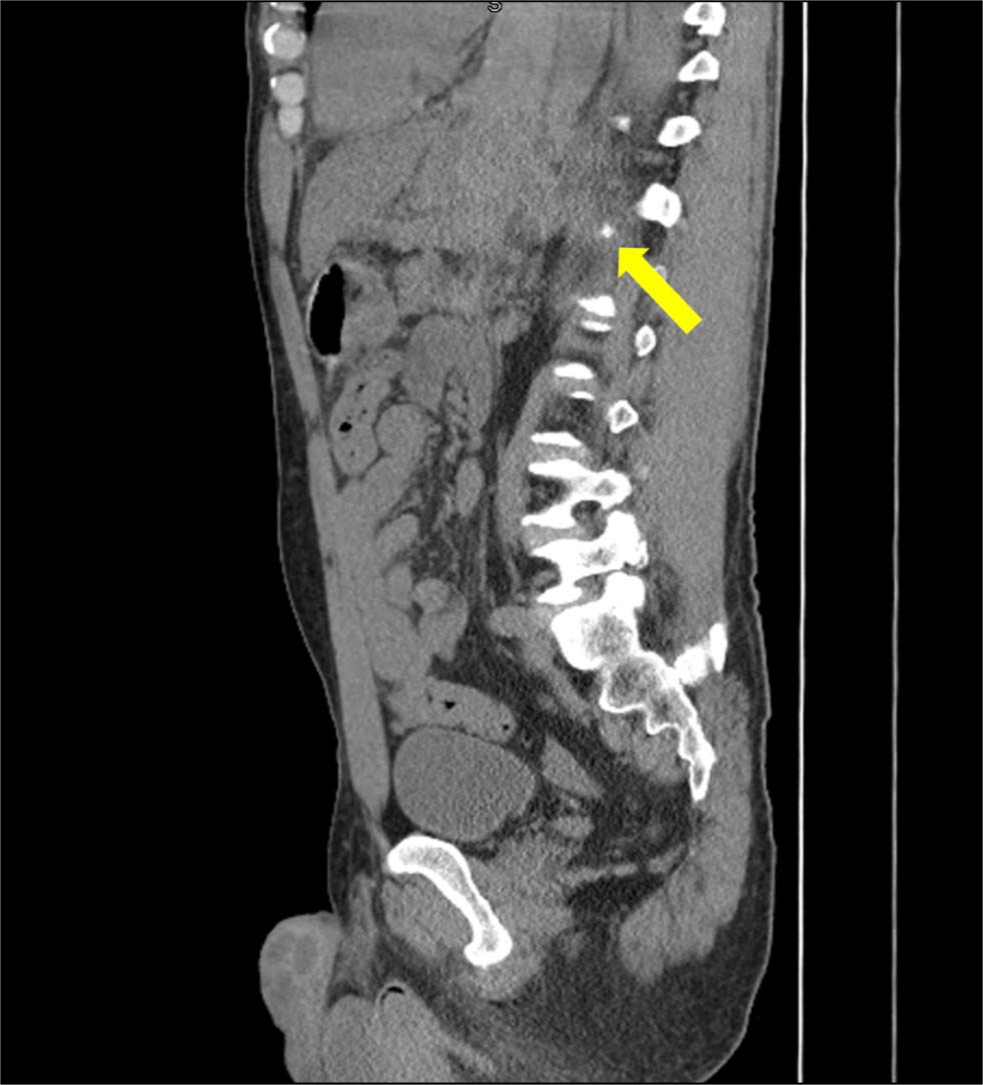
Noncontrast computed tomography chest abdomen and pelvis sagittal view showed relatively higher density left pleural effusion (yellow arrow) and deep pelvic fluid.

**Figure 2.**
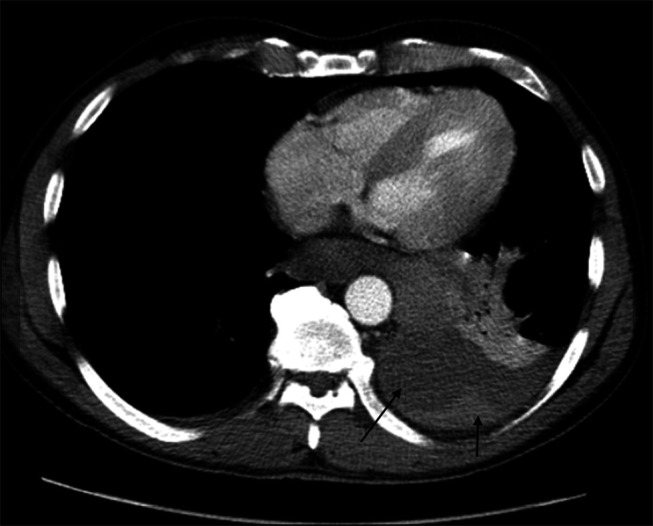
Computed tomography chest with contrast was performed which showed wall thickening of the mid-distal esophagus and gastric cardia with a large volume surrounding high-density material, favoring hematoma/hemorrhage, with a small focus of active contrast extravasation/bleeding medial to gastric cardia. Moderate left hemothorax is noted (black arrows).

A 50-year-old man presented with a 1-day history of worsening epigastric and left upper quadrant abdominal pain. Review of symptoms positive for unintentional weight loss of 20 lbs in the past 6 weeks. He denied any history of pancreatitis, blunt trauma, abdominal surgery, fever, chills, nausea, vomiting, or bowel or urinary changes. Other significant histories included hypertension, untreated hepatitis C infection, intravenous drug abuse, and smoking. The patient denied any recent alcohol use. On examination, he was afebrile, heart rate of 92 per minute, blood pressure of 150/113 mm hg, respiratory rate of 18 per minute, and saturating 96% on room air. The patient had diminished breath sounds at the left lung base. He had abdominal tenderness in the epigastric and left upper quadrant areas without peritoneal signs. Laboratory workup revealed a white cell count of 18,700 cells/cu mm, hemoglobin 13.6 g/dL (14.1 g/dL 2 months prior), platelets 222,000 cells/cu mm, prothrombin time 14 seconds, international normalized ratio 1.2, sodium 135 mEq/L, potassium 4.6 mEq/L, chloride 102 mEq/L, glucose 135 mg/dL, blood urea nitrogen 21 mg/dL, creatinine 1.15 mg/dL, total bilirubin 0.7 mg/dL, alanine transaminase 120 U/L, aspartate transaminase 80 U/L, alkaline phosphatase 71 U/L, troponin I 0.014 ng/mL, and procalcitonin 0.11 ng/mL. The urine drug screen was positive for methamphetamine and fentanyl. Hepatitis C antibody was positive; hepatitis A immunoglobulin M equivocal/gray zone, HIV and coronavirus disease 2019 negative. Noncontrast computed tomography (CT) chest abdomen and pelvis sagittal view showed relatively higher density left pleural effusion and deep pelvic fluid (Figure 1). CT chest with contrast was performed which showed wall thickening of the mid-distal esophagus and gastric cardia with a large volume surrounding high-density material, favoring hematoma/hemorrhage, with a small focus of active contrast extravasation/bleeding medial to gastric cardia. Moderate left hemothorax was noted. Small-volume, high-density fluid in the left upper quadrant tracking along the greater curvature of the stomach, most compatible with hemoperitoneum is observed (Figure 2).

**Figure 3.**
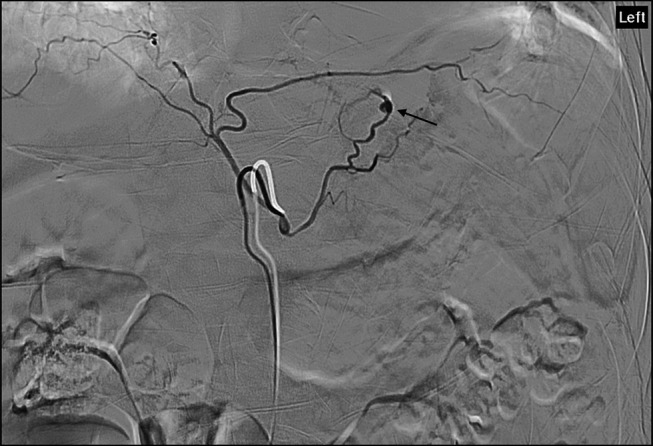
Inferior phrenic artery angiogram showing a pseudoaneurysm (black arrow) of the left inferior phrenic artery with contrast extravasation.

A corroborating history of the present illness and the newly discovered posterior mediastinal lesion raised clinical suspicion for varied differential diagnoses such as possible malignancy, esophageal perforation, bleeding varix, and left-side hemothorax. Interventional radiology consultation was obtained, and the patient underwent a celiac, left gastric, splenic, multilevel intercostal, and IPA angiogram. A pseudoaneurysm of the left IPA with contrast extravasation was identified (Figure 3). Coil and gel foam embolization of IPA was successfully performed. Further during the hospitalization, he underwent esophagogastroduodenoscopy revealing esophageal candidiasis, and there were no signs of esophageal fistula or tears. The gastroesophageal junction was nodular, and the biopsy showed junctional mucosa with mild chronic inflammation and reactive changes, negative for intestinal metaplasia, dysplasia, and malignancy. Thoracentesis was performed which showed the left hemothorax. Pleural fluid studies were negative for neoplasm. The patient was treated with fluconazole for candidiasis.

## DISCUSSION

A review of literature showed only a few cases of IPA aneurysms.^4,9^ We believe ours is the first case report that presented as a posterior mediastinal lesion extending into the abdomen.

**Table:** 

Causes of inferior phrenic artery pseudoaneurysm
Complicated acute or chronic pancreatitis Postendovascular interventions Post-surgeries like gastrectomy, coarctectomy, etc. Trauma Vasculitis (Polyarteritis nodosa, Takayasu arteritis, Henoch Schoenlein Purpura, Bechet disease) Collagen vascular diseases (Ehlers-Danlos syndrome, Marfan syndrome) Sepsis (mycotic aneurysm) Segmental arterial mediolysis

Aneurysm formation occurs in approximately 40%–90% of polyarteritis nodosa patients due to immune complex deposition.^11^ We excluded vasculitis from our differentials because we had normal inflammatory markers and no other symptoms. Ehlers-Danlos syndrome is caused by defective collagen formation (COL5A genes), which results in defective vessel formation and aneurysms. However, splanchnic vasculature involvement is extremely rare,^12^ and our patient has no clinical features or family history of collagen vascular disease. Our patient had no history of trauma, surgery, or pancreatitis. Sepsis can cause mycotic aneurysms, but our patient's blood cultures were negative.

**Figure 4.**
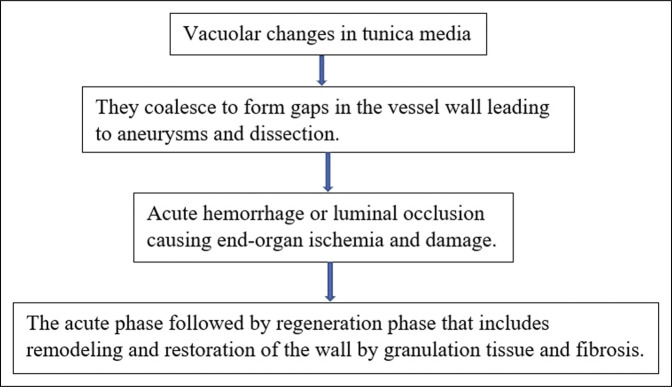
Pathophysiology of segmental arterial mediolysis.

We ruled out alternate etiologies based on the history, clinical examination, laboratory, and radiological findings. Given the patient's history of hypertension, we suspect the cause of the pseudoaneurysm is SAM, which is associated with vascular stress and damage. SAM is an idiopathic noninflammatory, nonatherosclerotic vascular disease.^13^ Hypertension is noted in 43% of cases diagnosed with SAM.^13^ However, diagnostic criteria for SAM still need to be validated. The pathophysiology is described in Figure 4. The splanchnic arteries are most involved in SAM seen in middle- to old-aged people.^14^ Of the splanchnic arteries, celiac trunk involvement is seen in 70%–80% of cases.^10^ The presentation can be asymptomatic or with nonspecific symptoms like abdominal pain, flank pain, hypotension, and melena. The symptoms are attributed to acute or chronic bleeding into the gastrointestinal tract or into the peritoneum or the retroperitoneum.^15^

The rupture of an aneurysm may occur spontaneously or after exertion. Ruptured IPA pseudoaneurysm is diagnosed by clinical assessment combined with radiological investigations. If management is delayed, the rupture could lead to 100% mortality.^16^ Contrast-enhanced angiography is the first line of investigation due to its non-invasive nature and its benefit of providing an idea of the endovascular management plan. Pseudoaneurysms appear at the time of arterial enhancement and are contiguous and eccentric to the vessel lumen. They are seen as an abnormal contrast collection on axial CT imaging. Magnetic resonance angiography is preferred in cases with contrast allergies and renal failure; however, it is not the foremost investigation as it is expensive and takes time. Catheter angiography remains the gold standard in evaluating and as a therapeutic modality for pseudoaneurysms.^17^ It assists in prompt diagnosis and management with a high rate of patient survival.^16^

Embolization is done in cases with active extravasation or if the aneurysm is in the accessible blood vessel.^15^ Endovascular intervention with a combination of gelatin sponges, coils, stents, and liquid polymer embolization is the first line of management.^18^ The selection of embolization material and technique depends on the location and accessibility of the aneurysm. In challenging anatomy, combined coil embolization with a stent is considered.^18^ N-butyl cyanoacrylate (cast forming agent) and coils are used during endovascular intervention to occlude the bleeding or aneurysmal arteries.^10^ Managing hypertension is also critical given its potential contribution to the pathology. Surgical intervention has a higher rate of postoperative morbidity and mortality.^15^ Obtaining a histopathological specimen from the involved blood vessel would support the suspected diagnosis; however, this has not been performed on our patient.

So far, the literature has reported 12 cases of IPA aneurysms (2 reports) and pseudoaneurysms (10 including ours) caused by a variety of factors such as acute or chronic pancreatitis, post-surgery, and trauma.^4,7–9,17,19–24^ Of the 12 patients, 5 developed IPA aneurysms after surgery, 3 after pancreatitis, 1 after blunt trauma, and 2 spontaneously, with varying complications. However, ours is the first case report of a ruptured IPA pseudoaneurysm caused by SAM.

IPA pseudoaneurysm can be due to various causes. Although an uncommon cause, SMA can cause aneurysms due to long-standing hypertension contributing to vascular stress and damage. The initial diagnosis is based on the patient's history, clinical examination, and laboratory and radiological findings. It could present as mass compressing surrounding structures or hemorrhagic shock or as a well-contained hematoma causing hypotension. The initial step is imaging and emergency management with angiography. The endovascular intervention using suitable delivery materials like coils, or gel foam plays a vital role and decreases the higher mortality rate associated with ruptured aneurysm. Management of hypertension is also crucial. The specific diagnosis can be attained by histopathological examination after resection or postmortem.

## DISCLOSURES

Author contributions: All authors contributed, reviewed, and edited the manuscript.

Financial disclosure: The authors declare that no funds, grants, or other support were received during the preparation of this manuscript. The authors have no relevant financial or non-financial interests to disclose.

Informed consent was obtained for this case report.
